# Patterns of human gene expression variance show strong associations with signaling network hierarchy

**DOI:** 10.1186/1752-0509-4-154

**Published:** 2010-11-12

**Authors:** Kakajan Komurov, Prahlad T Ram

**Affiliations:** 1 Department of Systems Biology, The University of Texas M.D. Anderson Cancer Center, 7435 Fannin St, Houston, TX 77054 USA

## Abstract

**Background:**

Understanding organizational principles of cellular networks is one of the central goals of systems biology. Although much has been learnt about gene expression programs under specific conditions, global patterns of expressional variation (EV) of genes and their relationship to cellular functions and physiological responses is poorly understood.

**Results:**

To understand global principles of relationship between transcriptional regulation of human genes and their functions, we have leveraged large-scale datasets of human gene expression measurements across a wide spectrum of cell conditions. We report that human genes are highly diverse in terms of their EV; while some genes have highly variable expression pattern, some seem to be relatively ubiquitously expressed across a wide range of conditions. The wide spectrum of gene EV strongly correlates with the positioning of proteins within the signaling network hierarchy, such that, secreted extracellular receptor ligands and membrane receptors have the highest EV, and intracellular signaling proteins have the lowest EV in the genome. Our analysis shows that this pattern of EV reflects functional centrality: proteins with highly specific signaling functions are modulated more frequently than those with highly central functions in the network, which is also consistent with previous studies on tissue-specific gene expression. Interestingly, these patterns of EV along the signaling network hierarchy have significant correlations with promoter architectures of respective genes.

**Conclusion:**

Our analyses suggest a generic systems level mechanism of regulation of the cellular signaling network at the transcriptional level.

## Background

Gene expression changes in the cell allow for reprogramming of cellular behavior depending on the extracellular conditions. Global gene expression profiling of cells has become a routine procedure in biology, and extensive work has been done in the recent years studying gene expression programs under various conditions [1-4]. In addition, many aspects of gene expression behavior at the DNA and chromatin level have also been identified [5-9]. Although these studies yielded much insight into the regulation of gene expression under the specific conditions studied, we do not have a clear understanding of global patterns in gene expression regulation in human cells in response to extracellular stimuli. Some notable studies addressing functional aspects of gene expression regulation at a systems level have been performed in yeast [10-14], however, an analysis of general trends in the gene expression response of human cells to extracellular cues and of their functional consequences on the regulation of human cell behavior has not been performed.

We undertook a functional analysis of global trends in the expression variance of human genes in response to extracellular cues. Expression variance of a gene can be defined as the frequency and magnitude of change in its mRNA levels in response to changing extracellular conditions and can be thought of as *regulatability *of a gene at the mRNA level. First, we report that human genes display a wide spectrum of EV under physiological conditions, with some genes showing very little variation in their mRNA levels, while some have extremely variable expression across a wide range of conditions. The EV pattern of genes strongly correlates with their promoter architecture, such that genes with lowest EV have open promoters with constitutive RNA polymerase occupancy, while those with highest EV have closed promoters with little or no RNA polymerase occupancy. Then, we show that this pattern of EV under physiological conditions reflects positioning of genes in the hierarchy of cell signaling, such that the most highly regulated genes are located at the apical parts of signaling hierarchy and are generally functionally more specialized. Finally, we discuss implications of these findings on our understanding of the generic mechanisms of regulation of cell behavior as it relates to restructuring of the intracellular protein interactome. This study uncovers some of the basic principles of transcriptional response in human cells and expands our understanding of conditional gene expression at the protein network level suggested by earlier studies on tissue-specific gene expression [15].

## Results

### Calculating Expression Variance of human genes

**Figure 1:**
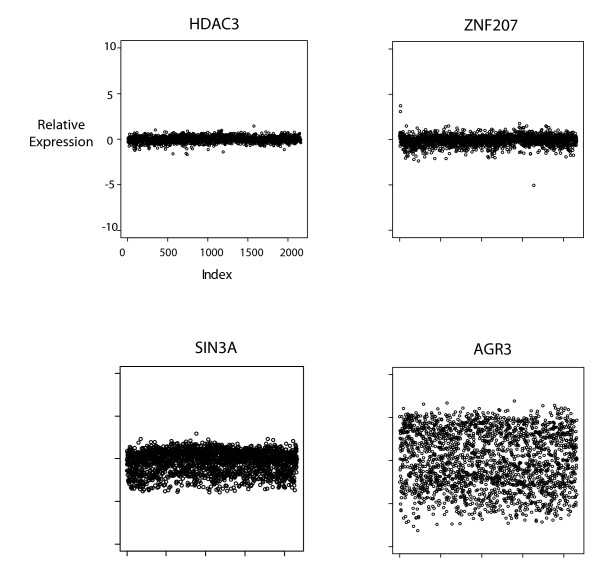
**Plots of expression patterns of genes with EV_expo _values within each quartile of the EV_expo _distribution**. The x-axes indicate samples, and y-axes indicate relative expression levels (see Methods for array normalizations).

In order to calculate global patterns of expression variance (EV) of human genes, we used the extensive collection of human cancer tissue microarrays of the Expression Project for Oncology (ExpO) of the International Genomics Consortium (http://www.intgen.org/expo), which contains expression microarray profiles of 2158 tumor tissue samples. This dataset contains samples dissected from diverse tissues with various types of cancer with different characteristics and treatments, and therefore spans a wide spectrum of cellular environments. We calculated EVs for each human gene by taking statistical variance of normalized expression levels of each gene across the whole dataset. Normalization of expression levels of each gene was done by first normalizing the samples in the dataset by quantile normalization [16], so that each sample in the dataset has an identical distribution, and then dividing the expression level of the gene in each sample by the median of its expression level across all samples, which resulted in a measure of deviation of the expression levels of each gene from their median. Representative plots of expression profiles of some low and high EV genes are shown in Figure 1. Varying levels of expression variation is evident between high and low EV genes. Expression levels of genes with low EV seem to be relatively stable regardless of extracellular conditions, while some genes seem to have extremely variable expression pattern across many different conditions. This suggests that some genes may be preferentially regulated during cellular adaptation to its environment, while some genes are generally not regulated at a transcriptional level.

It is possible that the EV values simply reflect basal tissue specific expression variations of genes and not the variability of their expression under different cellular conditions. In order to test this, we calculated tissue-specific EVs of genes using only samples in the ExpO dataset collected from breast, lung or colon, thereby obtaining EVs of genes for each individual tissue type. If the EVs reflect tissue-specific expression variations of genes, there should not be high correlation between tissue-specific EVs. However, there is a high correlation between breast and lung tissue-specific EVs (Additional File 1). Similarly high correlation was also observed between ovary and lung tissue EVs (Additional file 2, which indicates that EV mostly reflects variability of genes between different cellular and extracellular conditions rather than tissue-specific expression patterns of genes. We also tested correlation of EV values between different probes of the same gene, and find similar high correlation (Spearman's ρ = 0.45, n = 10,263, *P *<<10^-16^), indicating that the EV values identified here represent gene-specific variations of mRNA levels.

### EV reflects gene regulation under varying extracellular conditions

To further confirm that our EV values reflect cellular response to varying extracellular conditions rather than being an artifact of tissue samples, we compiled an independent collection of microarray gene expression datasets from 14 different studies measuring responses of cultured human cells to various receptor ligands (EGF, heregulin, TGF-beta, TNF-alpha, interleukin 1, FGF2, arachidonic acid, thrombin, leukotriene, estradiol and sphingosine) (CK dataset, see Methods). We normalized each microarray sample in the dataset by their corresponding controls (i.e. no treatment conditions) and discarded the control samples, so that each sample in the CK dataset reflects fold-changes in response to the corresponding stimulus. Therefore, this dataset contains measurements of gene expression change in various human cell lines under 149 different stimulation conditions. The expression variance of genes calculated using the CK dataset is in a significantly high agreement with the EV values calculated using the ExpO dataset of 2158 tissue samples (Spearman's ρ = 0.69, see Additional file 3). Since EV_CK _values reflect fold changes of gene expression upon a large number of different stimulations, our observation indicates that EV_expo _values reflect true expression variations of genes within cells under different extracellular environments, rather than an artifact of tissue- or cell type-specific expressional variations.

### EV is not an artifact of mRNA abundance

Total mRNA expression levels of genes are extremely variable (spanning almost 4 logs), and this can substantially contribute to the variability of genes between different conditions. Indeed, EV of genes has a significant negative correlation with their average expression levels across the whole ExpO dataset (Spearman's ρ = -0.59 for EV_expo _and -0.53 for EV_CK_), so that genes with low mRNA abundance are more likely to have variable expression. Therefore, it is possible that our observations above and below simply reflect the correlations of total expression levels of gene mRNAs rather than their variability. In order to test this, we calculated partial correlation between EV_expo _and EV_CK _having controlled for average mRNA expression levels of genes and find that the correlation strength between these EV values calculated from different datasets is still significantly strong (partial Spearman's ρ = 0.58), indicating that the observed EV is not an artifact of mRNA abundance. In order to confirm this observation, we selected genes with similar average mRNA levels (300 < average expression < 350, n = 831), and tested if the correlation between EV_expo _and EV_CK _is still high. Indeed, although the correlation of total mRNA levels with either EV_CK _or EV_expo _is lost (Spearman's ρ values of 0.02 and -0.003, respectively), the correlation between EV_CK _and EV_expo _is still significantly high (Spearman's ρ = 0.50, Additional file 4), which strongly suggests that expression variability is an intrinsic characteristic of genes rather than an artifact of their total mRNA abundance. Importantly, the correlations we present below are also reproducible having controlled for the total mRNA levels of genes (see below and Additional file 5).

### EV reflects RNA Polymerase II promoter occupancy

**Figure 2:**
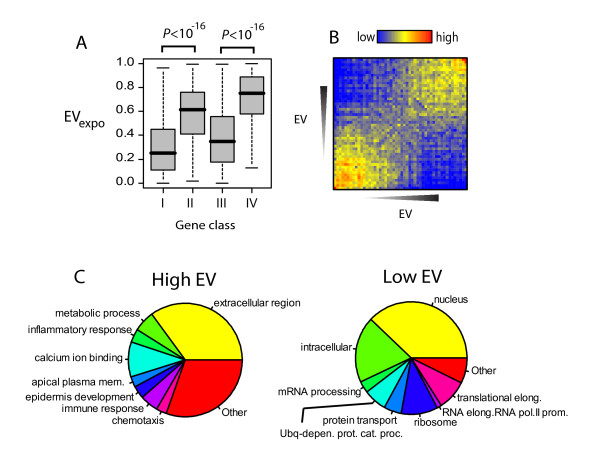
**Functional significance of Expression Variance of human genes**. A) Boxplots of EV_expo _values of genes in the classes of genes as defined in Kim et al (2005) [8]. Class I of genes have RNA Polymerase PIC occupancy and active transcription, Class II has PIC occupancy but no active transcription, Class III have no or low (transient) PIC occupancy but active transcription, Class IV have no detectable PIC occupancy or transcription (see Kim et al (2005)). P-values of difference were calculated using Wilcoxon rank sum test. B) Heatmaps of interaction densities between low and high EV_expo _genes based on functional relations. Each square *i,j *represents density of interactions between bins *i *and *j *as defined by number of interactions between bins *i *and *j *divided by total number of interactions of bins *i *and *j *in the network. C) Pie charts of most significantly enriched GO categories in 500 highest and lowest EV_expo _genes. Significance of enrichment was assessed by hypergeometric distribution (*P *< 10^-5^). Only GO categories with more than 5 annotated genes were selected.

Next, we asked if EVs of genes correlates with the pattern of their promoter activities. Kim et al (2005) conducted a comprehensive study mapping active promoters across the human genome and identified 4 classes of genes based on their expression and RNA polymerase II pre-initiation complex (PIC) promoter occupancy [8]. The first class (Class I) of genes had PIC occupancy and increased histone acetylation in their promoters and were actively transcribed. The second class (Class II) had PIC occupancy, however were not actively transcribed. The third class of genes were actively transcribed although no PIC could be detected, while the fourth class (Class IV) had no PIC occupancy or detectable transcript levels and had reduced histone acetylation at their promoters [8]. We find that there is a high concordance between EV values and these gene classes (Figure 2A), which was also reproduced with EVs of the CK dataset (Additional file 6). Low-EV genes mostly belong to classes I and III, reflecting constitutive and high promoter activity, whereas high-EV genes mostly belong to classes II and IV, which are mostly expressed in a condition-specific manner. This is concordant with the invariant constitutive expression pattern of low EV genes and highly variant condition-specific expression pattern of high EV genes. These observations suggest that low EV genes are generally highly active and abundantly transcribed, while high EV genes are transcribed in a condition-specific manner.

### Functional distinction of genes based on EV

We have previously shown organization of genes into separate modules based on their expression variation in yeast [10,12]. We wanted to determine if expression variation in human genes has a functional significance similar to that observed in yeast. In order to answer this question, we constructed a comprehensive network of human genes based on their functional similarity, where each interaction is between two genes sharing a significant functional annotation from either Gene Ontology [17] or KEGG [18] (the Fun-Net, see Methods). Then, we tested whether subnetworks of genes with specific functional associations segregated based on their EV. In order to gain a comprehensive view of gene-gene association preferences in the Fun-Net based on their EV, we binned genes into 50 bins based on their EV and calculated interaction preferences between each bin pair in the Fun-Net. As expected, the heatmap of interaction preferences shows a clear clustering of low and high EV genes into separate functional categories (Figure 2B). This is not an artifact of the network connectivity, as this pattern is not observed in a network where node positions have been randomly shuffled (Additional file 7). Similarly analysis using different bin sizes did not significantly alter the outcome (Additional file 8). A similar high correlation and interaction preference pattern, albeit weaker, is observed when protein-protein interaction network is used for gene-gene interactions instead of the Fun-Net (Additional file 9). These observations show that human genes can be functionally separated based on their EV patterns. Low overall association of genes with low and high EV genes in either network suggests that the cellular functions performed by the low and high EV genes are distinct, similar to what we have shown for yeast.

Next, in order to see which cellular functions are represented by the high and low EV genes, we calculated relative enrichment of the top and bottom 500 genes within the EV distribution for specific GO functional categories. Figure 2C shows pie-charts of most enriched (hypergeometric distribution p-value < 10^-5^) functional categories in 500 genes with lowest or highest EV. Genes encoding for cellular functions pertaining to cellular homeostasis: mRNA transcription and processing, protein synthesis and proteasomal protein degradation, are the most significantly enriched functional categories among genes with lowest EV (Figure 2C). However, genes exhibiting highest EV are mainly composed of genes encoding proteins in the extracellular space, including extracellular matrix (ECM) components, growth factors and extracellular proteases. A similar pattern is identified using the EV_CK _values (Additional file 10), where the values reflect fold inductions of genes within the same cell line in response to a treatment. Therefore, the differential enrichment of high and low EV genes for, respectively, extracellular space and intracellular homeostasis genes reflects biological pattern of cellular response to extracellular conditions.

### EV of signaling genes reflects their role in the signaling hierarchy

**Figure 3:**
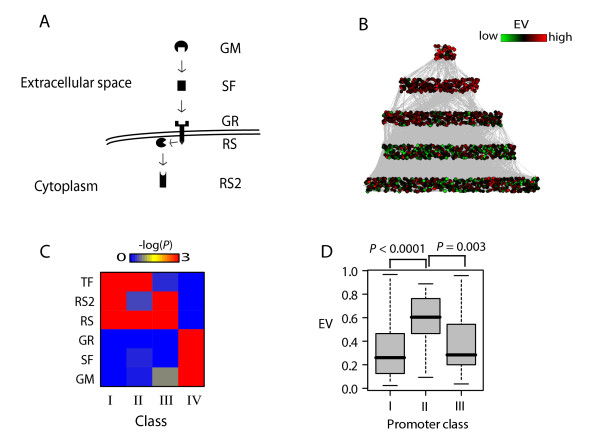
**Pattern of EV along the signaling hierarchy**. A) A visual depiction of the signaling hierarchy as defined in text and Methods. B) Network plots of the signaling hierarchy. Each layer corresponds to the corresponding layer in A. Node colors show EV_expo _values of corresponding genes and lines indicate directed protein-protein interactions. C) Heatmap of enrichment p-values of each gene class as defined in Kim et al (2005) for genes in corresponding signaling hierarchy levels. Colors indicate negative log (base 10) of p-values as determined by hypergeometric distribution formula. D) Boxplots of EV values of RS level genes with class I, II and III promoters. P-values of difference were calculated by Wilcoxon rank sum test.

Based on the observations above, we reasoned that extracellular ligands for cellular transmembrane receptors may be more variable than their receptors, meaning that cells are more likely to modulate the expression levels of secreted factors rather than their receptors in response to extracellular cues. We compared EVs of genes annotated as "receptor binding" (GO:0005102), "growth factor activity" (GO:0008083) or "cytokine activity" (GO:0005125) *and *"extracellular space" (GO:0005615) (SF list, n = 269 genes) to those annotated as "transmembrane receptor activity" (GO:0004888), "receptor activity" (GO:0004872) *and *"plasma membrane" (GO:0005886) (GR list, n = 1038 genes) (see Additional file 11). Although EVs of both classes are significantly higher when compared to the rest of genes, EVs of the SF list are significantly higher than those of the GR list (see Figure 3B). We wanted to determine if EVs of genes involved in signal transduction reflect the hierarchical position of the corresponding signaling molecules within the signaling network. In order to answer this question, we compiled a comperehensive signaling network from online databases (5499 genes and ~22,000 interactions, see Methods), and defined 5 levels of signaling hierarchy based on the positions of the signaling molecules (Figure 3A). The first level, growth factor modulators (GM class), are secreted molecules that modulate the activities of receptor-binding secreted factors. This class includes genes such as SFRP2 (Secreted Frizzled-Related Protein 2, regulator of WNT proteins), MMP1 (matrix metaloprotease 1, regulator of various growth factors/cytokines), IGFBP1 (IGF binding protein 1) and LTBP1 (latent TGF-beta binding protein). The next two levels are secreted factors (SF) and receptors (GR), explained above. Receptor substrates (RS) are molecules immediately downstream of receptors (GR), such as G-proteins (GNA genes), receptor-associated kinases (e.g. IRAK genes, ADRBK2, JAK1), and adaptor proteins (e.g. GRB2, SOS1-2, FADD, IRS1) among others; and the next class are molecules that mediate signal transduction downstream of RS (RS2) (see Methods). Strikingly, EV patterns of these levels display a gradient, with the GM level being the most variable, and RS being the least variable among these hierarchy levels (Figure 3B). This pattern is also reproduced with EV_CK _values (Additional file 10). This suggests that transcriptional regulation of intracellular signaling pathways mostly happens at the level of secreted growth factor modulators and growth factors, while signaling molecules immediately downstream of signaling receptors seem to be the least transcriptionally modulated. Accordingly, the RS and RS2 levels are mostly found in class I through III of genes based on their PIC occupancy, while GM, SF and GR levels are in class IV (Figure 3C).

Interestingly, class II, which represents genes with PIC occupancy but no detectable transcription, is enriched for intracellular signaling genes in the RS level, not secreted factors, although this class has significantly high EV (see Figure 2A). This may indicate that class II contains condition-specific intracellular signaling genes, while classes I and III are enriched for constitutively expressed intracellular signaling genes. Indeed, genes with class II promoters contain high EV genes of the RS level, while classes I and III contain the low EV genes of the RS level (Figure 3D). Importantly, these observations suggest not only that genes coding for extra- and intra-cellular proteins can be distinguished based on their promoter architecture, but also that promoters of intracellular proteins among themselves are distinguished based on whether they are constitutive or condition-specific. In addition, while genes for condition-specific extracellular proteins are located within densely packed hypo-acetylated regions, condition-specific intracellular genes have relatively open promoters with a pre-assembled PIC. This suggests that regulation of transcription of genes coding for extracellular proteins may be fundamentally different from those coding for intracellular signaling genes (see Discussion).

### EV reflects functional centrality

**Figure 4:**
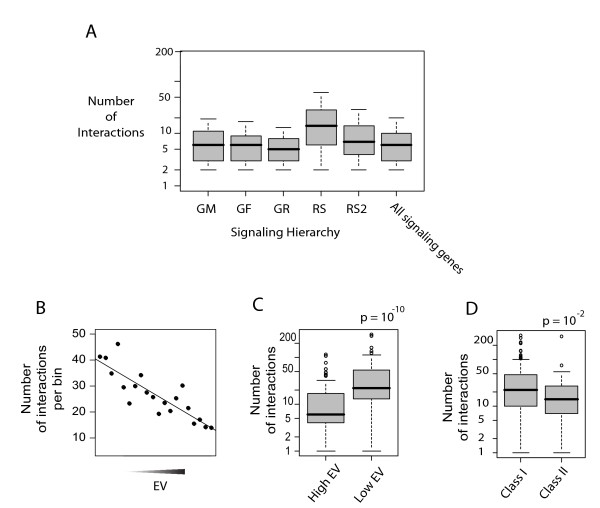
**Functional centrality of low-EV signaling genes**. A) Numbers of protein-protein interactions within each signaling hierarchy. B) Plot of average number of interactions of the RS and RS2 level genes depending on their EV. RS and RS2 level genes were arranged in the order of increasing EV and binned into 20 bins. C) Boxplots of numbers of protein-protein interactions of RS and RS2 level genes with high (>0.9) and low (<0.1) EV genes. D) Boxplots of RS and RS2 level genes with Class I and Class II promoter architecture. Y-axes in C and D are on a log scale. P-values show p-values of difference calculated by Wilcoxon test.

**Figure 5:**
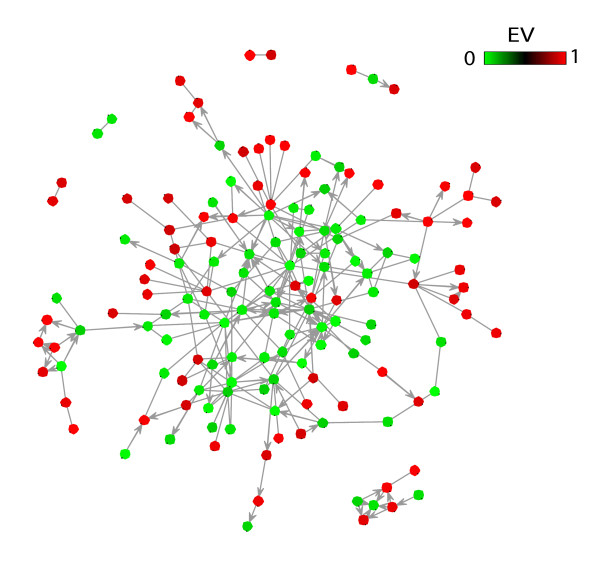
**Network of interactions of RS and RS2 level genes with high (>0.9) and low(<0.1) EV**. Colors of nodes indicate EV according to the color key.

Next, we asked whether the correlation of EV patterns of signaling genes with their positioning within the signaling hierarchy has a biological significance in terms of regulation of signaling pathways. Secreted factors are very specific in terms of their signaling targets (e.g. the chemokine CXCL12 specifically activates the G-protein coupled receptor CXCR4) therefore variation of their levels through transcriptional regulation may provide a high level of specificity in the regulation of signaling pathways. However, the receptor substrates are utilized by many signaling pathways (e.g. G-proteins are utilized by a large variety of G-protein coupled receptors), and therefore variation in their expression can lead to major rearrangements in the signaling architecture of the cell. Therefore, it is possible that transcriptional regulation of signaling in response to extracellular cues involves highly selective activation/inhibition of specific pathways, rather than involving large rearrangements of the signaling network. In order to test this, we compared total number of protein-protein interactions within each hierarchical class. Since the functions of signaling molecules mainly involve protein-protein interactions, the total number of protein-protein interactions of a signaling protein may provide an estimate of the number of different processes/functions that it can be involved in. Indeed, the RS class has the most overall number of interactions among all the classes (Figure 4A). In order to see if lower EV of most central proteins is a general trend in the intracellular protein interaction network, we correlated total number of interactions of the RS and RS2 proteins with their EVs. There is a significant negative correlation of EVs of RS and RS2 proteins and their number of protein interactions (Figure 4B, Spearman's ρ = -0.27, n = 1189, *P *< 10^-20^), which supports our hypothesis that differential expressional variation of genes within the signaling hierarchy can at least in part be explained by their functional centrality in the signaling network. Moreover, the RS and RS2 level proteins with high EV (EV > 0.9) have significantly less number of protein-protein interactions than those with low EV (EV < 0.1) (Figure 4C). In addition, the intracellular signaling genes with Class II promoters, which are mainly condition-specific (see Figure 3D), have significantly less number of protein-protein interactions than those with Class I promoters (Figure 4D), which are mainly constitutive (see Figure 3D). In order to get a view of the layout of proteins with different EVs within the signaling network, we plotted the signaling network of proteins of the RS and RS2 levels with high and low EVs. This network in Figure 5 shows a clear differential distribution of high and low EV proteins. While proteins with low EV are mainly located in the central dense regions of the network, those with high EV are mainly located at the periphery and generally have sparser connections. These observations support our hypothesis that condition-specific genes encode proteins with less central roles in the signaling network. The finding that genes with lowest EVs mostly comprise genes involved in the cellular homeostatic processes (transcription, translation, etc...), which can be regarded as the most central cellular processes, also adds to the hypothesis that mRNA levels of genes with highest functional centrality in the cell are modulated less and the most variable genes are those encoding more specialized regulatory proteins.

## Discussion & Conclusions

### Expressional variation of human genes

Computational studies in yeast combining large-scale gene expression data with protein interaction networks have revealed high level of modularity in the network with respect to transcriptional regulation [10,12,13,19,20]. However, with the exception of some recent studies [15,21], such studies with human data have not been performed. Here, we report a study of global patterns in the expressional variation of human genes across a wide spectrum of conditions, and the functional significance of EV with respect to the regulation of signaling network architecture. Our findings were reproduced using two independent data compendiums, suggesting that these observations reflect true biological relationships. In addition, since variations in mRNA levels of genes have been shown to be in a relatively high agreement with corresponding variations in protein levels [22-24], the patterns of EV discovered in this study give insight into the patterns of regulation of signaling networks in response to extracellular stimuli.

Our results show that human genes are extremely variable in the extent of regulation of their mRNA levels. While some genes' mRNA levels are highly variable across many conditions, some show very tight expression patterns with very little variation. As expected, genes with lowest EV are those involved in cellular "housekeeping" functions, such as mRNA synthesis and processing as well as protein synthesis and degradation. In agreement with prior data about condition-specific genes [8,25], genes with high EV mainly have "covered" promoters with reduced histone acetylation and no RNA polymerase pre-initiation complex (PIC) occupancy, while genes with low EV have high PIC occupancy and increased histone acetylation in their promoters.

### Transcriptional regulation of intracellular and extracellular proteins

Our analyses correlating previous classification of genes into 4 distinct classes of promoters by Kim *et al *(2005) [8] revealed that there is a high concordance of EV values with their promoter architectures. Low EV genes are abundantly and actively transcribed, while high EV genes are generally not active. Most interestingly, high EV genes coding for intracellular signaling proteins have acetylated promoters with pre-assembled PIC, while high EV genes coding for extracellular proteins have hypo-acetylated promoters without PIC. Importantly, this may imply that the regulation of gene expression for extracellular proteins involves chromatin remodeling and PIC assembly, while that for intracellular proteins occurs at the level of RNA polymerase II elongation, rather than PIC assembly and chromatin remodeling. It has been reported that promoter-proximal pausing of the RNA polymerase II and its subsequent release for elongation is a major mechanism of regulation of human gene expression [26], which suggests that this mechanism may be employed for the regulation of intracellular proteins.

It should be noted that the study of Kim *et al *was performed on human fibroblasts, and therefore it could be argued that the classification of genes into distinct promoter classes may be specific to fibroblasts, despite the observed high correlation of the EV patterns with these classes. We find that EV of genes is highly similar between different tissues (ExpO dataset) and different conditions (CK dataset), suggesting that a common pool of condition-specific genes may exist, selective modulation of which may drive cell adaptation. Similarly, genes with lowest EV are primarily those with housekeeping functions in the cell, and are therefore likely to be expressed in all cell types. Therefore, it is likely that the overall chromatin architecture of most human promoters is also largely conserved between different cell types, and tissue and cell type-specific promoters may constitute a relative minority. This hypothesis is not far-fetched, as another recent study analyzing chromatin architecture around gene promoters in a number of different human cell lines reported more than 70% similarity in observed positioning of nucleosomes in promoters of different cell types [27].

### Regulation of signaling network architecture

The observation that genes regulated most in response to extracellular stimuli are secreted factors and their receptors implies that regulation of cell behavior mostly involves modulation of the composition of the extracellular environment. Even the intracellular signaling proteins with high EV seem to be mainly those with specialized roles in the regulation of signaling and with fewer number of functional interactions. This indicates that the repertoire of the extracellular space and of their receptors mostly determines cell behavior, while the intracellular signaling hubs are mainly common for different cell types/conditions. Since in a scale-free network, such as the protein-protein interaction network [28], highly connected hubs play an important role in determining the overall architecture [29], our findings may suggest that the overall architecture of the signaling network is relatively stable across different conditions. Therefore, regulation of cell signaling during cell adaptation is mainly at the level of signaling inputs at the extracellular space, and minor highly specific rearrangements within the intracellular network. This in turn suggests that relatively same signaling network architecture allows for integration of various inputs to elicit a variety of cell fates, reminiscent of a multifunctional electronic circuit. A relatively stable network architecture where the hubs are involved in multiple processes may be evolutionarily more advantageous over a highly dynamic network architecture where hubs are condition-specific. It is interesting to note that similar conclusions have been drawn from recent studies on tissue-specific genes, where it was reported that tissue-specific proteins are enriched for extracellular proteins [30], and another reporting that tissue-specific proteins generally have less number of protein interactions [15]. Therefore, it is possible that the regulatory principles in response to diverse external stimuli uncovered in this study also apply to tissue-specific modulation of cell behavior.

It can be argued that quantitating protein-protein interactions to show relative centrality of proteins may introduce artifacts of historically more studied proteins. However, we suggest it is a fair assumption that the distribution of well-studied proteins across the EV spectrum is relatively uniform so as to allow for the detection of statistically significant patterns.

## Methods

### Datasets

The ExpO dataset was downloaded from the web site for Expression Project for Oncology (http://www.intgen.org/expo/). Each column in the final dataset of 2158 samples was first normalized by quantile normalization, and then each row was normalized by its median value and log2 transformed. EV values were determined as statistical variance value of a gene across all the samples in the normalized dataset. The CK compendium was derived from datasets in Gene Expression Omnibus: GDS649 (IL1 treatment of HUVEC cells), GDS1290 (TGF-beta treatment of Th1 and Th2 cells), GDS1249 (arachidonic acid treatment of dendritic cells), GDS2516 (interferon treatment of endothelial and fibroblast cells), GDS3215 (retinoic acid treatment of sebocyte cells), GDS1926 (leukotriene and thrombin treatment of endothelial cells), GDS2626 (EGF and HRG treatment of MCF7 cells), GDS2422 (FGF2 treatment of fibroblasts), GDS2484 (TNF-alpha treatment of endothelial cells), GDS2622 (EGF treatment of MCF10A cells), GDS3217 (estradiol treatment of MCF7), GDS2090 (sphingosine treatment of glioblastoma cell line), GDS855 (TGF-beta treatment of CD34+ cells) and GDS854 (TGF-beta treatment of a leukemia cell line). The columns in each dataset in the CK compendium were normalized by their respective control conditions (e.g. 0 time point), and columns for control conditions were discarded. Values were log2 transformed and each column was then normalized to have a mean of 0 and a variance of 1. EV values for each data compendium is given in Additional file 12.

### Networks

Functional similarity interactions (Fun-Net) were constructed using Gene Ontology (GO) annotations as defined in the Entrez Gene database, and also metabolic pathway annotations in the KEGG database. Any two genes sharing a metabolic pathway annotation from KEGG were assigned an interaction. In the case of GO annotations, two genes were assigned an interaction if the overlap of their GO annotations was significant compared to the rest of the genes: *s_ij _*= **|∩ ***G_k_***|/***n*, where *s_ij _*is the significance of overlap between genes *i *and *j*; *G_k _*is the set of genes that have the GO term *k*, where *k *belongs to the set of GO terms common to genes *i *and *j*, and *n *is the total number of genes. If *s_ij _*< 0.001, genes *i *and *j *were assigned an interaction. Protein-protein interactions were compiled from online databases HPRD [31], BIND [32], HomoMINT [33], Gene [34] and IntAct [35]. For the signaling network, we compiled signaling interactions from KEGG, BioCarta (http://pid.nci.nih.gov/) and TRANSPATH [36], as well as through manual curation of some undirected protein-protein interactions. Transcription factor-target interactions were obtained from ORegAnno [37], TRANSFAC [38] and interactions in BIND classified as protein-DNA. Both networks are available from authors upon request.

### Signaling hierarchy

Cell surface receptors and extracellular proteins were determined by combining genes with GO annotations as described in text. Receptors were assigned directly to GR. SF class was defined by determining extracellular proteins with direct signaling interactions with the GR group proteins. GM is defined as extracellular proteins with direct signaling interactions with the SF but not GR groups. RS are proteins with direct signaling interactions with GR and RS2 are those with direct signaling interactions with RS but not GR. Lists of genes within each hierarchy class is given in Additional file 13.

## Authors' contributions

KK designed and performed the analyses and wrote the manuscript. PR contributed to the design of experiments and writing the manuscript. Both authors read and approved the final manuscript.

## Supplementary Material

Additional file 1 **Additional Figure 1**. Plot of correlation of EV values of genes calculated using only tissue samples from breast and lung. Click here for file

Additional file 2 **Additional Figure 2**. Plots of EV values calculated using tissues from only ovary vs. colon (n ~ 19,000). P-value of correlation is < 10^-300 ^(Spearman's rank correlation). Click here for file

Additional file 3 **Additional Figure 3**. Plot of correlation of EV_expo _and EV_CK _values of genes. Click here for file

Additional file 4 **Additional Figure 4**. Plot of correlation of EV_expo _and EV_CK _values of genes with average expression levels between 300 and 350. Click here for file

Additional file 5 **Additional Figure 5**. Correlation of EV with the positioning of genes on the signaling hierarchy or promoter classes is not an artifact of their expression levels. A-C) Genes with expression levels between 1000 and 1500 were selected. Box plots of A) their expression levels within each promoter class. Their EVs within B) each promoter class and C) signaling hierarchy class are shown. D-F) Same as in A-C, but with genes with expression levels greater than3000. Note that even for genes with different ranges of expression levels the EV's of the promoter class and signaling hierarchy exhibit the same distribution pattern (B, C, E, F). Click here for file

Additional file 6 **Additional Figure 6**. Boxplot of EV_CK _values of genes within each promoter class. P-values were calculated by Wilcoxon rank sum test. Click here for file

Additional file 7 **Additional Figure 7**. Heatmap of interaction preferences in the original (left) and a randomized network (right). Randomized network was generated by randomly shuffling node positions keeping the network structure same. Click here for file

Additional file 8 **Additional Figure 8**. Same as in Figure 2B, but with 100 bins. Click here for file

Additional file 9 **Additional Figure 9**. Heatmap of protein-protein interaction densities between genes with different EV. Click here for file

Additional file 10 **Additional Figure 10**. Boxplots of EV_CK _values of genes within each signaling hierarchy. P-values were calculated by Wilcoxon rank sum test. Click here for file

Additional file 11 **Additional Figure 11**. Boxplots of EV_CK _values of genes classified under given Gene Ontology terms. Numbers above the boxes indicate number of genes within each category. P-values were calculated by Wilcoxon rank sum test. Click here for file

Additional file 12 **Additional Table 1**. EV_expo_, and EV_CK _values of genes. Click here for file

Additional file 13 **Additional Table 2**. List of genes within each signaling hierarchy. Click here for file
