# Supplementary figures and images for: Patterns of human gene expression variance show strong associations with signaling network hierarchy
Source: BMC Syst Biol. 2010 Nov 12;4:154. doi: 10.1186/1752-0509-4-154 (PMC2992512; doi:10.1186/1752-0509-4-154)

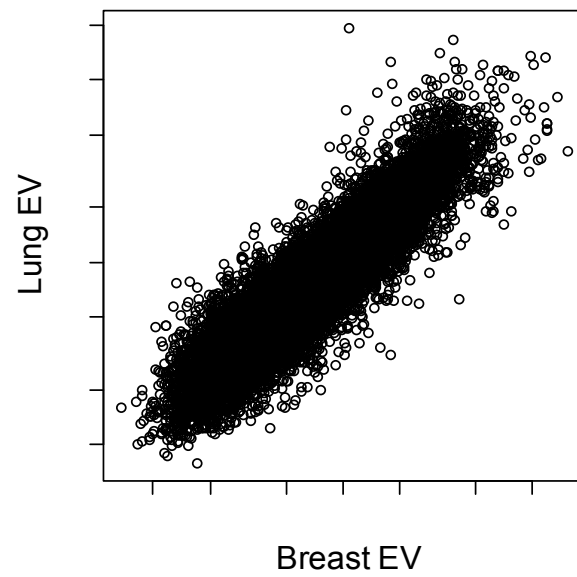

Supplement: Additional file 1 — Additional Figure 1. Plot of correlation of EV values of genes calculated using only tissue samples from breast and lung. [file 1752-0509-4-154-S1.PDF]

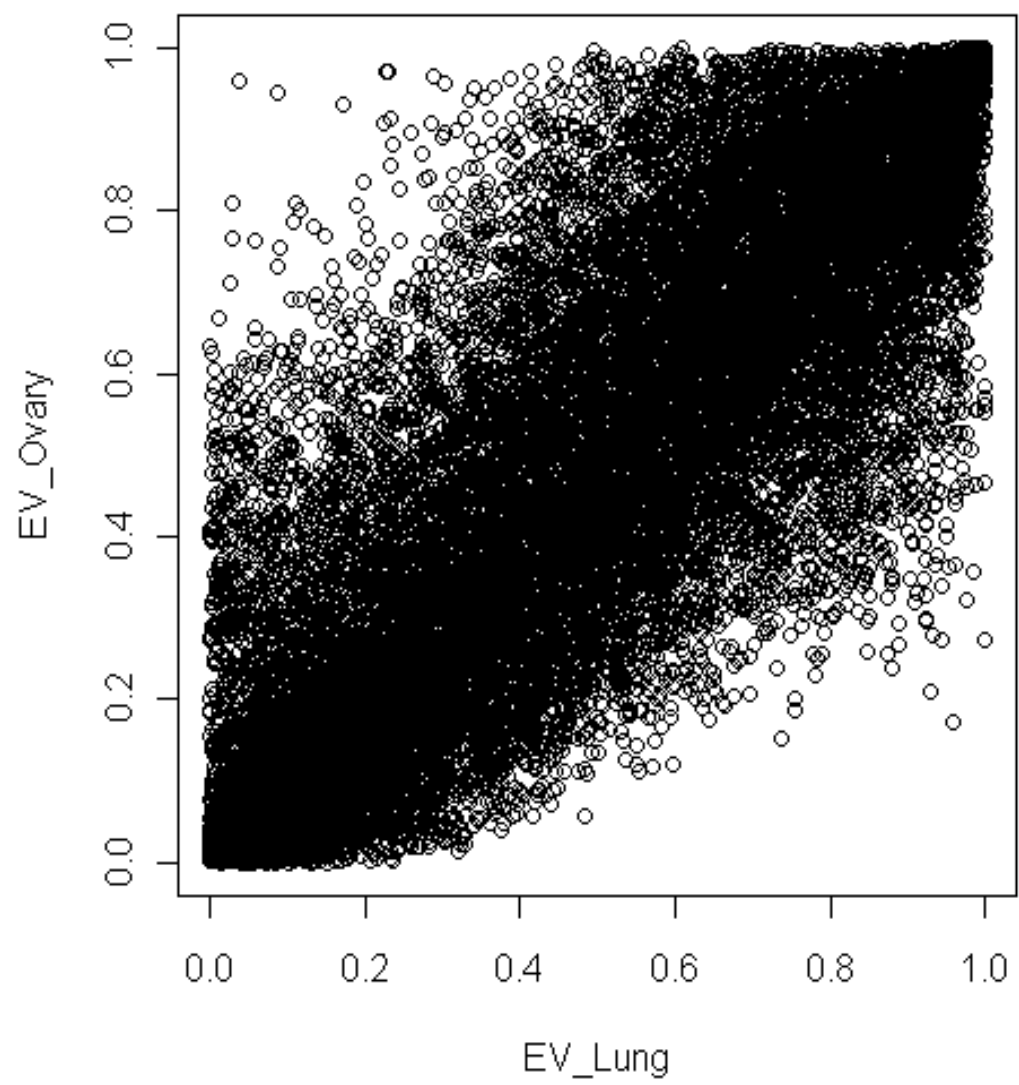

Supplement: Additional file 2 — Additional Figure 2. Plots of EV values calculated using tissues from only ovary vs. colon (n ~ 19,000). P-value of correlation is < 10-300 (Spearman's rank correlation). [file 1752-0509-4-154-S2.PDF]

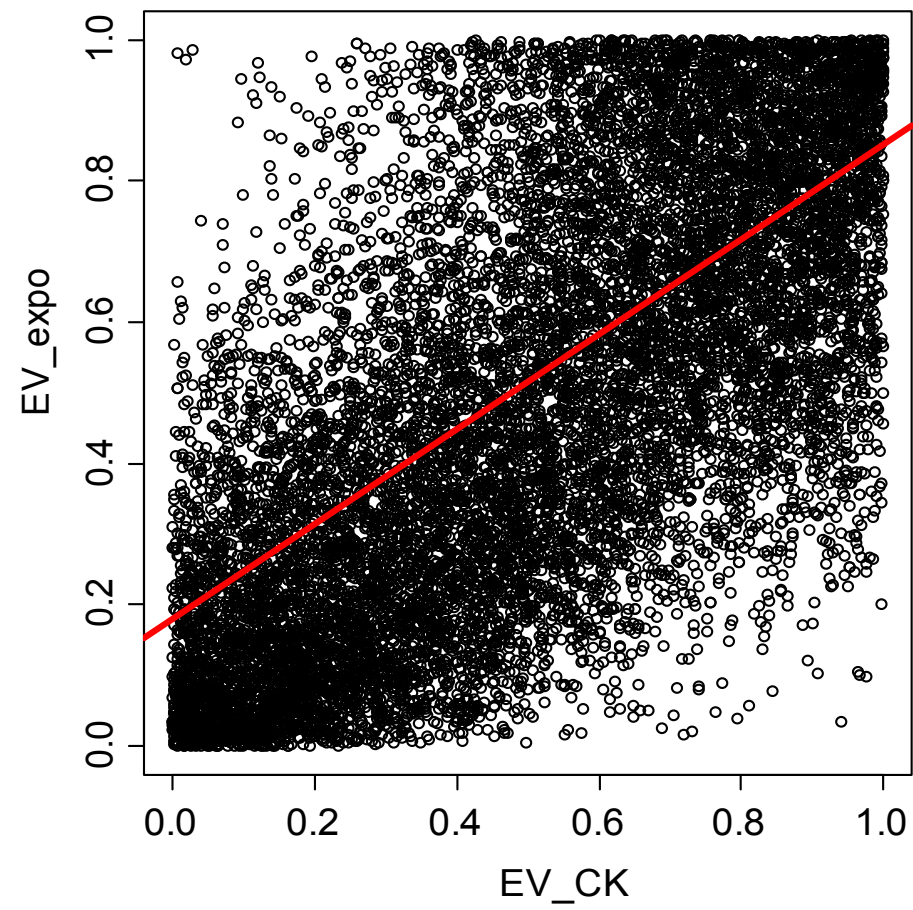

Supplement: Additional file 3 — Additional Figure 3. Plot of correlation of EVexpo and EVCK values of genes. [file 1752-0509-4-154-S3.PDF]

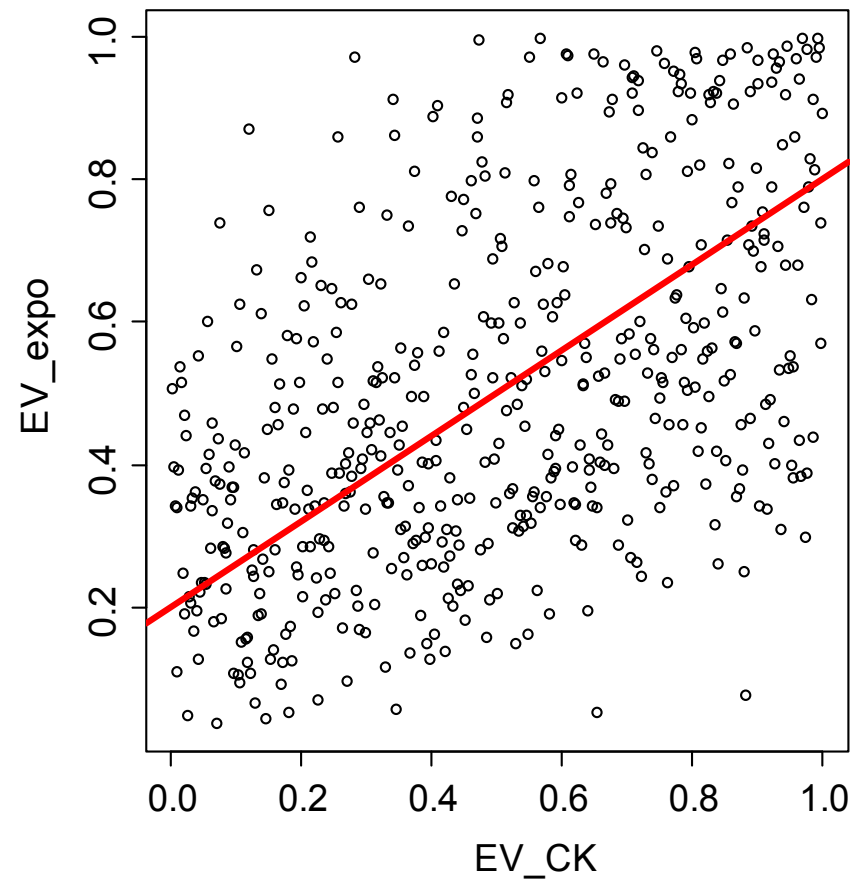

Supplement: Additional file 4 — Additional Figure 4. Plot of correlation of EVexpo and EVCK values of genes with average expression levels between 300 and 350. [file 1752-0509-4-154-S4.PDF]

**A**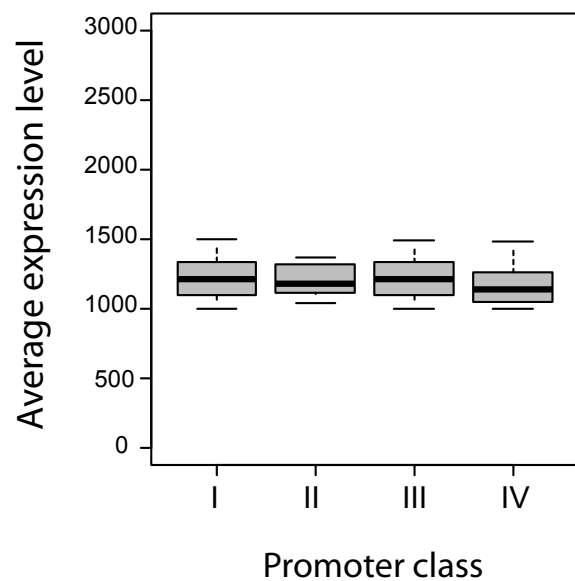**B**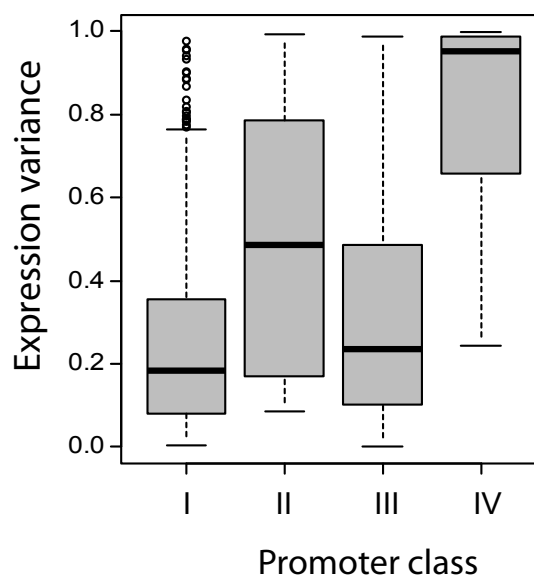**C**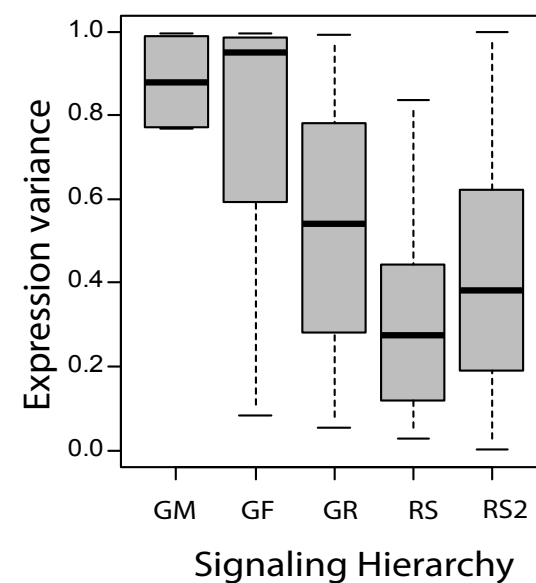**D**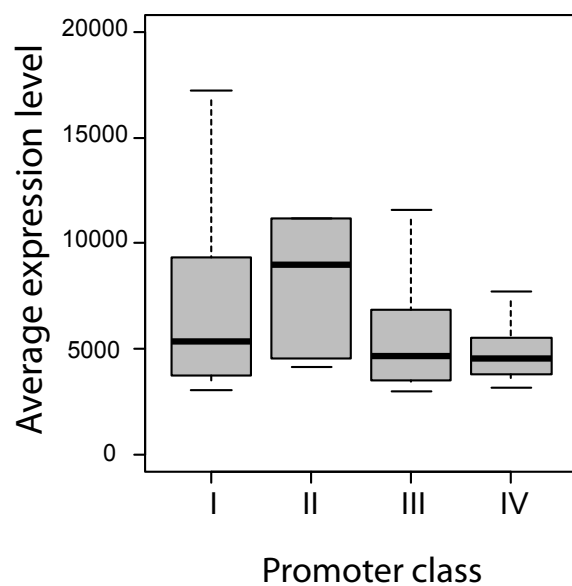**E**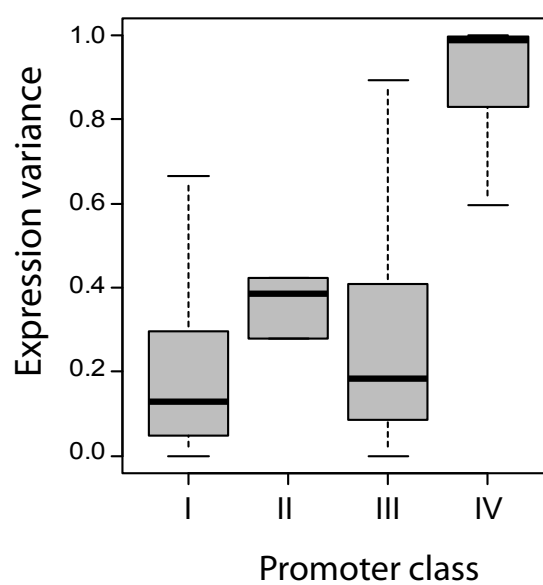**F**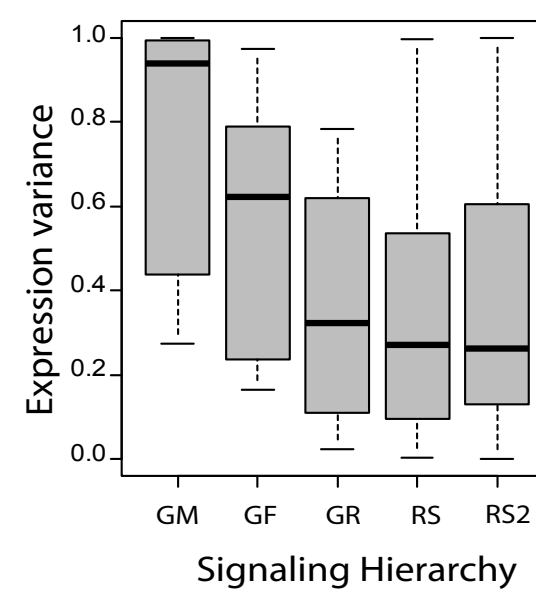

Supplement: Additional file 5 — Additional Figure 5. Correlation of EV with the positioning of genes on the signaling hierarchy or promoter classes is not an artifact of their expression levels. A-C) Genes with expression levels between 1000 and 1500 were selected. Box plots of A) their expression levels within each promoter class. Their EVs within B) each promoter class and C) signaling hierarchy class are shown. D-F) Same as in A-C, but with genes with expression levels greater than3000. Note that even for genes with different ranges of expression levels the EV's of the promoter class and signaling hierarchy exhibit the same distribution pattern (B, C, E, F). [file 1752-0509-4-154-S5.PDF]

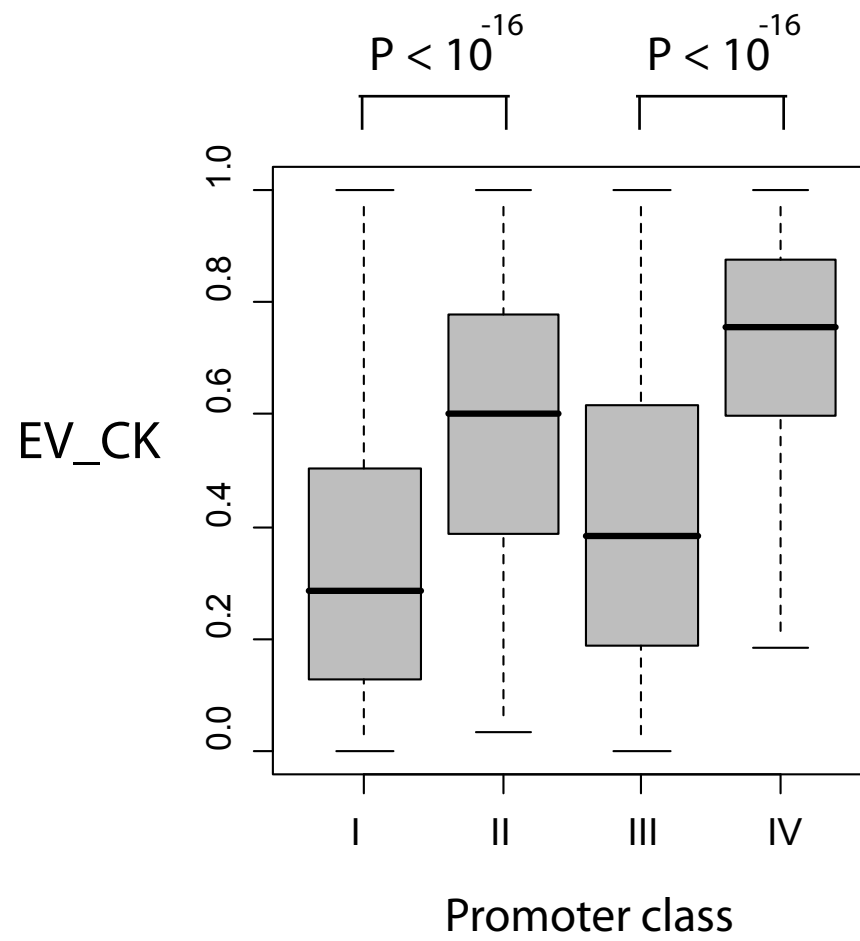

Supplement: Additional file 6 — Additional Figure 6. Boxplot of EVCK values of genes within each promoter class. P-values were calculated by Wilcoxon rank sum test. [file 1752-0509-4-154-S6.PDF]

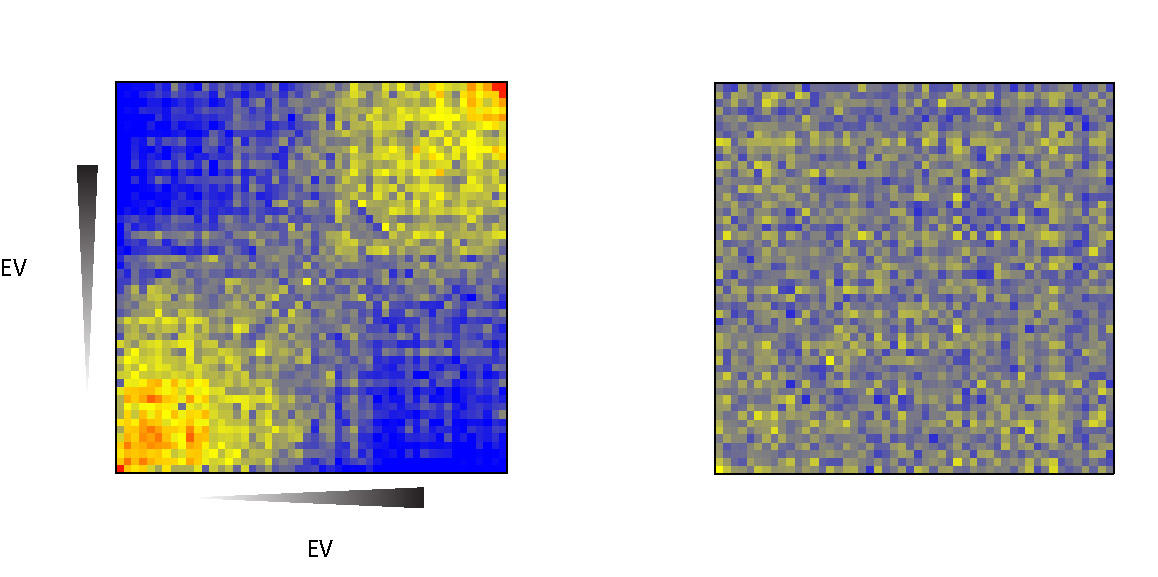

Supplement: Additional file 7 — Additional Figure 7. Heatmap of interaction preferences in the original (left) and a randomized network (right). Randomized network was generated by randomly shuffling node positions keeping the network structure same. [file 1752-0509-4-154-S7.JPEG]

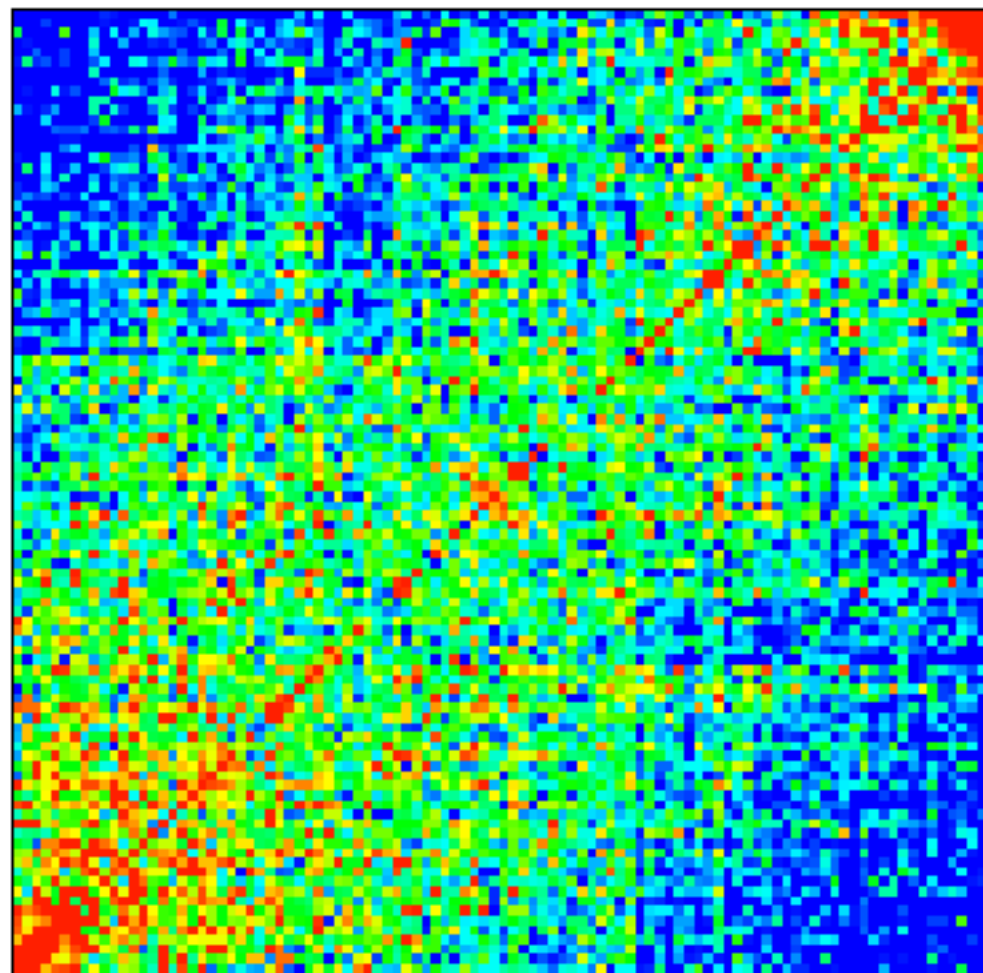

Supplement: Additional file 8 — Additional Figure 8. Same as in Figure 2B, but with 100 bins. [file 1752-0509-4-154-S8.PDF]

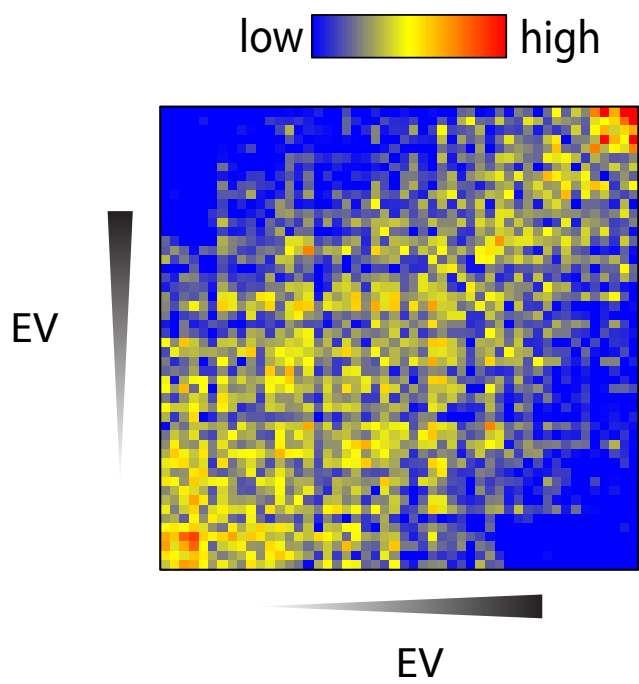

Supplement: Additional file 9 — Additional Figure 9. Heatmap of protein-protein interaction densities between genes with different EV. [file 1752-0509-4-154-S9.PDF]

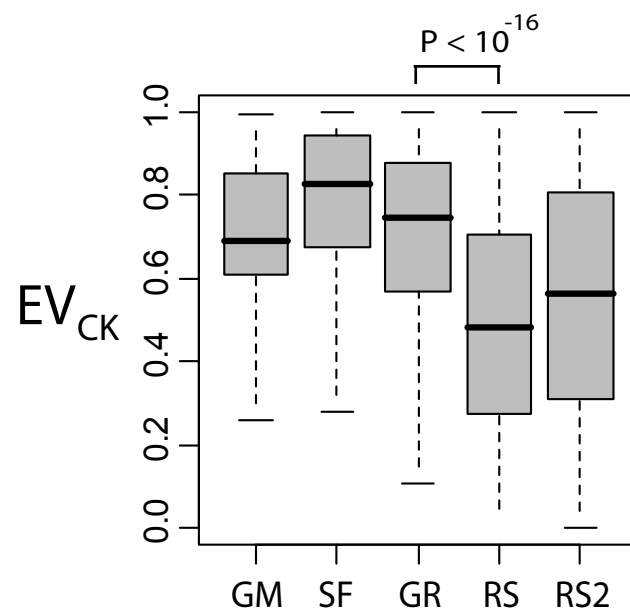

Supplement: Additional file 10 — Additional Figure 10. Boxplots of EVCK values of genes within each signaling hierarchy. P-values were calculated by Wilcoxon rank sum test. [file 1752-0509-4-154-S10.PDF]

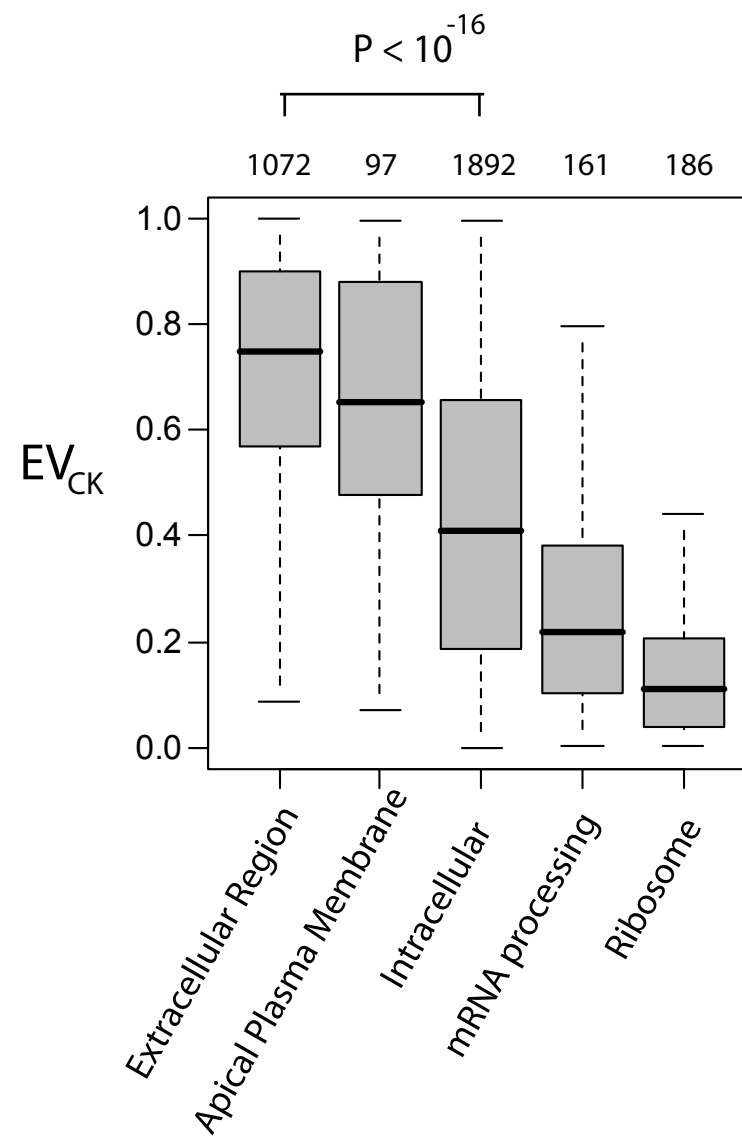

Supplement: Additional file 11 — Additional Figure 11. Boxplots of EVCK values of genes classified under given Gene Ontology terms. Numbers above the boxes indicate number of genes within each category. P-values were calculated by Wilcoxon rank sum test. [file 1752-0509-4-154-S11.PDF]
